# Free Radicals in Adolescent Varicocele Testis

**DOI:** 10.1155/2014/912878

**Published:** 2014-12-14

**Authors:** Carmelo Romeo, Giuseppe Santoro

**Affiliations:** ^1^Department of Pediatric, Gynaecological, Microbiological and Biomedical Sciences, A.O.U. “G. Martino”, University of Messina, Via Consolare Valeria, 98125 Messina, Italy; ^2^Department of Biomedical Sciences and Morphofunctional Images, A.O.U. “G. Martino”, University of Messina, Via Consolare Valeria, 98125 Messina, Italy

## Abstract

We examine the relationship between the structure and function of the testis and the oxidative and nitrosative stress, determined by an excessive production of free radicals and/or decreased availability of antioxidant defenses, which occur in the testis of adolescents affected by varicocele. Moreover, the effects of surgical treatment on oxidative stress were provided. We conducted a PubMed and Medline search between 1980 and 2014 using “adolescent,” “varicocele,” “free radicals,” “oxidative and nitrosative stress,” “testis,” and “seminiferous tubules” as keywords. Cross-references were checked in each of the studies, and relevant articles were retrieved. We conclude that increased concentration of free radicals, generated by conditions of hypoxia, hyperthermia, and hormonal dysfunction observed in adolescent affected by varicocele, can harm germ cells directly or indirectly by influencing nonspermatogenic cells and basal lamina. With regard to few available data in current literature, further clinical trials on the pre- and postoperative ROS and RNS levels together with morphological studies of the cellular component of the testis are fundamental for complete comprehension of the role played by free radicals in the pathogenesis of adolescent varicocele and could justify its pharmacological treatment with antioxidants.

## 1. Introduction

Free radicals, produced in both physiological and pathological conditions in mammalian tissues [1], are chemical species highly unstable because of the presence in their structure of one or more unpaired electrons; for this reason, they are very reactive and seek to reach a more stable state by coupling with other molecules or atoms. They constitute a wide family of compounds and can be divided into two groups: Reactive oxygen and nitrogen species (ROS and RNS, resp.) that principally include superoxide anion radicals, hydroxyl radicals, hydrogen peroxide (H_2_O_2_), peroxynitrite and other peroxides, and nitric oxide [2].

During normal physiologic processes, ROS/RNS perform numerous roles in regulation of cellular function by interacting with cellular lipids, proteins, nucleic acids, and carbohydrates. In pathological conditions, nuclear and mitochondrial membranes, structural and cytoplasmic proteins, complex carbohydrates, deoxyribonucleic acid (DNA), and ribonucleic became targets of disproportionate levels of free radicals [3–6].

They are generated by several cellular components: mitochondria, peroxisomes, smooth endoplasmic reticulum, cytoplasm, nucleus, and plasma membranes [7].

Their potentially toxic effect is prevented by antioxidants, both enzymatic and nonenzymatic, that dispose, scavenge, and suppress the excessive formation of free radicals or oppose their actions [3–5]. The major antioxidant enzymes expressed in the testis are superoxide dismutase, catalase, and glutathione/glutathione dismutase [8–11]. The efficacious nonenzyme antioxidant factors in reducing testicular oxidative stress are vitamins C and E, melatonin, and resveratrol [12–15]. Also spermatozoa are filled out with antioxidant defense mechanisms and are likely to quench ROS, thereby protecting gonadal cells and mature spermatozoa from oxidative damage [16].

When an excessive production of ROS and RNS and/or decreased availability of antioxidant defenses occur, the oxidative and nitrosative stress (O and NS) takes place, inducing tissue damage in several pathologies [2, 17–24].

In the male reproductive tract, physiological quantities of ROS play important roles on sperm function, regulating capacitation, acrosome reaction, hyperactivation, and the fusion of spermatozoa with the oocyte [7, 25]. In particular conditions, O and NS may play a detrimental role for male fertility, such as toxicant exposure, chemotherapy, ionizing radiation, orchitis/inflammation, varicocele, cryptorchidism, aging, and testicular torsion [5].

Varicocele, the varicosity of the pampiniform venous plexus of the spermatic cord, represents the first cause of male infertility [7]. In the adolescent, its incidence is 12,4–17,8%, with an average of 14,7% [26]. Considering that once a varicocele has developed, it persists in adulthood [27], its surgical treatment, aside from the classical indications, is important to arrest the progressive damage that inevitably threatens for several years the adolescent testis [28].

## 2. Nitric Oxide and Adolescent Varicocele

Our group has demonstrated, for the first time, NO overproduction within dilated spermatic veins in adolescent affected by varicocele [29]. The NO level was established by determination of serum concentration of L-hydroxyarginine (L-NHA), a NO synthase by-product, and of nitrite/nitrate (NO_*x*_) end-product of NO metabolism. The concentration of L-NHA was increased by 50-fold in the spermatic vein when compared with the peripheral vein. When the adolescents were stratified for age at operation and time of onset of symptoms, no correlation was demonstrated with the concentration of NO in the spermatic vein [29]. The NO_*x*_ levels in the serum of varicocele patients were measured using a fluorometric assay that has been reported to be 50 to 100 times more sensitive than Griess reaction [30]. Similarly, a significant increase of NO_*x*_ in the spermatic vein was shown [29]. Moreover, the concentration of nitrotyrosine, a biological marker of NO-induced damage, was demonstrated to be significantly higher in the spermatic vein of adolescent with varicocele compared with the peripheral veins of the same patients, indicating that NO causes nitration of plasma proteins with higher molecular weight [31].

Subsequently, these results were confirmed by the study of Türkyilmaz, who showed, in adolescents with grades II and III varicocele, a significantly increase in the spermatic vein of NO_*x*_ and malondialdehyde (indicator of lipid oxidation as a result of increased ROS) (MDA) levels compared with the peripheral samples in the same patients [32].

NO is produced from the guanidine nitrogen of L-arginine by three isoforms of NO synthase (NOS): neuronal NOS (nNOS, NOS-1), first described in neurons, endothelial NOS (eNOS, NOS-3), first identified in endothelial cells, both of which are constitutive and calcium/calmodulin dependent isoforms, and the macrophage NOS (iNOS, NOS-2), an inducible calcium-independent isoform [33, 34].

ROS can be produced in large amounts by neutrophils and macrophages, but also by spermatozoa and other cells under pathological conditions [5, 35]. We have demonstrated that both adolescent normal and varicocele testis express eNOS at the level of vessels and Leydig cells. The iNOS was expressed in Leydig cells of normal testes and overexpressed in Leydig cells of varicocele testes [36].

**Figure 1:**
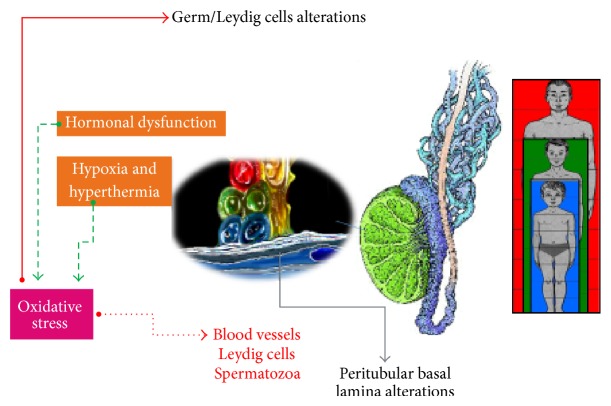
Oxidative stress generated by conditions of hypoxia, hyperthermia, and hormonal dysfunction observed in varicocele could have negative effects on germ and Leydig cells. Moreover, it could also have critical actions on blood vessels and spermatozoa of growing adolescents.

The seminiferous tubules include a complex stratified epithelium containing spermatogenic cells and supporting cells (Sertoli cells). The epithelium is surrounded by a lamina propria composed of a double-layered basal lamina and 5–7 external cellular layers, constituted by 3–5 inner layers of myofibroblasts and one or more outer layers of fibroblasts. These cellular layers are separated by laminae of extracellular connective tissue components. Between lamina propria and epithelium, a basal lamina exists. In the angular interstices between the seminiferous tubules, Leydig cells take place, which represent the endocrine component of the testis [36–38]. The NO produced by Leydig cells can freely diffuse across cellular membranes and stimulate testicular function at physiological concentrations. Similarly, its overproduction could promote different pathological actions as vasodilatation of blood vessels contributing to the blood stasis characteristic of varicocele and prolonged relaxation of myofibroblasts, in the context of the well-known effects of NO on smooth muscle cells observed in other tissues, consequently compromising the peristaltic activity necessary for sperm transport [34, 39]. Moreover, as testicular steroidogenesis [40] and germ cells [41] are limited by high levels of NO, perhaps maintaining the high oxidative stress, testosterone production by Leydig cells and germ cell function could be affected by the cytotoxic effects of NO in the long run. Similarly, in Sertoli cells, NO overproduction could alter both quantitatively and qualitatively the patterns of talin and vinculin, actin-associated proteins of the adherens junctions, known to be fundamental in regulating cellular adhesion, proliferation, migration, and differentiation [42–44]. Considering also the initial involvement of basal lamina in its two major components (collagen type IV and laminin) described in adolescents with varicocele [45] derives a possible general imbalance in interactions among Sertoli cells, peritubular myofibroblasts, Leydig cells, basal lamina, and germ cells due to NO (Figure 1).

## 3. Oxidative Stress and Spermatozoa

Recently, a positive relationship was demonstrated between semen parameters and ROS. Infertility is multifactorial phenomenon, with both men and women implicated in the cause [46]. The male factor is considered to be involved when one or more parameters as sperm concentration, motility, and/or morphology are abnormal [47]. Abnormalities in these parameters are called as oligozoospermia. In a cross-sectional study, compared with three control groups, oligozoospermic patients, with primary and secondary diagnosed infertility due to different pathologies including varicocele, in the oligoasthenoteratozoospermia, oligoteratozoospermia, and oligoasthenozoospermia groups, had significantly elevated ROS levels [46]. Moreover, infertile patients have been found to produce a greater amount of abnormal spermatozoa, which generate more ROS and less antioxidants, therefore leading to OS [48–50].

Free radicals affect spermatozoa in three main ways: membrane lipid peroxidation, DNA damage, and induction of apoptosis [51–54]. These cells, the first reported to show potential susceptibility to OS, are extremely sensible to oxidative insult because they lack the necessary cytoplasmic-enzyme repair systems [55]. This prerogative is principally due to the fact that their cell membranes are rich in polyunsaturated fatty acids, especially docosahexaenoic acid, rendering them highly exposed to oxygen-induced damage and hence lipid peroxidation (LPO) [56]. Subsequently, a quick loss of intracellular adenosine triphosphate from LPO determines axonemal damage, decreased sperm viability, and increased mid-piece sperm morphological defects, all of which contribute to decreased sperm motility as well as their ability to fertilize oocytes [52, 56, 57]. Furthermore, DNA fragmentation may impair the paternal genetic contribution to the developing embryo [48].

Another important effect of free radicals on spermatozoa is DNA damage [51, 52]. This occurs via direct attack on the purine and pyrimidine bases (especially guanine) and the phosphodiester backbones, hence destabilizing the DNA molecule and causing anomalies such as point mutations, polymorphisms, deletions, translocations, and even double-stranded breaks [7, 52, 54]. DNA fragmentation will cause abnormal fertilization, reduced implantation, and poor embryonic development such that the progeny is likely to have a shorter lifespan and an increased risk of developing cancer [7, 54, 58]. In adolescent patients with a clinical diagnosed bilateral grades II and III varicocele, an increase in sperm nuclear DNA fragmentation of class III (meaningful DNA fragmentation ) and class IV (high DNA fragmentation) has been shown when compared with the nonvaricocele group [59]. However, sperm DNA is less inclined to ROS-induced damage than the plasma membrane due to its highly condensed structure, which offers less surface area for attack by ROS [54, 58].

In cases of more severe damage, spermatozoa may undergo apoptosis, resulting in low sperm counts characteristic of idiopathic male factor infertility [51]. Sertoli cells can begin and regulate germ cell apoptosis through extrinsic mechanisms involving the interaction of Fas, a type I transmembrane protein, and its ligand, FasL, a type II membrane protein, both members of the tumor necrosis factor family [60]. When Fas binds to FasL, a molecular complex is formed, signaling initiation of apoptosis, which involves caspase 8 activation and a subsequent cascade of events leading to DNA fragmentation and cell death [61, 62]. In the testis, Fas is expressed in spermatogenic germ cells and FasL in Sertoli cells [60]. Apoptosis can be observed in spermatogonia, spermatocytes, and spermatids, all supported by Sertoli cells in seminiferous tubules [36, 63]. In sperm from 14 adolescents with varicocele grades II and III, using reverse transcription and real-time quantitative polymerase chain reaction, higher FasL mRNA levels have been demonstrated than in adolescents without varicocele. Moreover, these patients showed lower sperm concentration, when compared to the varicocele group. When submitted to correlation analysis, adolescents with varicocele presented a correlation between sperm concentration and FasL gene expression [64].

Finally, it has been shown that the level of seminal ROS is correlated with the grade of varicocele; the higher the grade of varicocele is, the greater the level of ROS detected is [65].

## 4. Effects of Surgical Treatment on Oxidative Stress

It has been demonstrated that surgical varicocelectomy in children aged 10 to 16 years with left-sided varicocele and ipsilateral testicular hypoplasia, performed by microsurgical inguinal ligation of the dilated spermatic veins with construction of a testicular-inferior epigastric venous shunt, reduces OS. One year after surgery the basal thiobarbituric acid reactive substances were reduced and the plasma peroxidation susceptibility (lag time) was increased, suggesting, respectively, a significant reduction of the basal peroxidative state and an improvement in the plasma resistance to free radical-induced damage or plasma antioxidant defenses [66].

On the contrary, no difference in levels of seminal products of lipid degradation (MDA) was observed in adolescents (14–19 years old) subjected to subinguinal microsurgical varicocelectomy, three months after surgery. It was supposed that varicocele repair was unable to alter the levels of seminal plasma oxidative stress because these levels were not elevated preoperatively. Anyway, in the same patients, the varicocelectomy was postoperatively associated with increased spermatozoa with intact nuclear DNA and mitochondrial activity [67].

## 5. Conclusions

This review has pointed out how adolescent testis, functionally characterized by high rates of metabolism and cell replication, is a structure particularly susceptible to the insult of O and NS in varicocele patients. From cellular component of seminiferous tubules, through basal lamina to spermatic veins, all elements are potentially victims of the imbalance between the production of ROS and RNS and the protective action of antioxidant system responsible for their neutralization and removal. Increased concentration of free radicals, generated by conditions of hypoxia, hyperthermia, and hormonal dysfunction observed in adolescent varicocele, can harm germ cells directly or indirectly by influencing nonspermatogenic cells and basal lamina (Figure 1). The higher the grade of varicocele is, the greater their seminal level is. With regard to few available data in current literature, further clinical trials on the pre- and postoperative ROS and RNS levels together with morphological studies of the cellular component of the testis are fundamental for complete comprehension of the role played by free radicals in the pathogenesis of adolescent varicocele and could justify its pharmacological treatment with antioxidants.
